# The effect of *Staphylococcus aureus* on innate and adaptive immunity and potential immunotherapy for *S. aureus*-induced osteomyelitis

**DOI:** 10.3389/fimmu.2023.1219895

**Published:** 2023-09-08

**Authors:** Yingqi Chen, Zixian Liu, Zexin Lin, Mincheng Lu, Yong Fu, Guanqiao Liu, Bin Yu

**Affiliations:** ^1^ Division of Orthopaedics and Traumatology, Department of Orthopaedics, Southern Medical University Nanfang Hospital, Guangzhou, China; ^2^ Guangdong Provincial Key Laboratory of Bone and Cartilage Regenerative Medicine, Southern Medical University Nanfang Hospital, Guangzhou, China; ^3^ Trauma Center, Department of Orthopaedic Trauma, The Second Affiliated Hospital of Hengyang Medical College, South China University, Hengyang, China

**Keywords:** *Staphylococcus aureus*, osteomyelitis, innate immunity, adaptive immunity, immune response

## Abstract

Osteomyelitis is a chronic inflammatory bone disease caused by infection of open fractures or post-operative implants. Particularly in patients with open fractures, the risk of osteomyelitis is greatly increased as the soft tissue damage and bacterial infection are often more severe. *Staphylococcus aureus*, one of the most common pathogens of osteomyelitis, disrupts the immune response through multiple mechanisms, such as biofilm formation, virulence factor secretion, and metabolic pattern alteration, which attenuates the effectiveness of antibiotics and surgical debridement toward osteomyelitis. In osteomyelitis, immune cells such as neutrophils, macrophages and T cells are activated in response to pathogenic bacteria invasion with excessive inflammatory factor secretion, immune checkpoint overexpression, and downregulation of immune pathway transcription factors, which enhances osteoclastogenesis and results in bone destruction. Therefore, the study of the mechanisms of abnormal immunity will be a new breakthrough in the treatment of osteomyelitis.

## 1 Introduction

**Figure 1:**
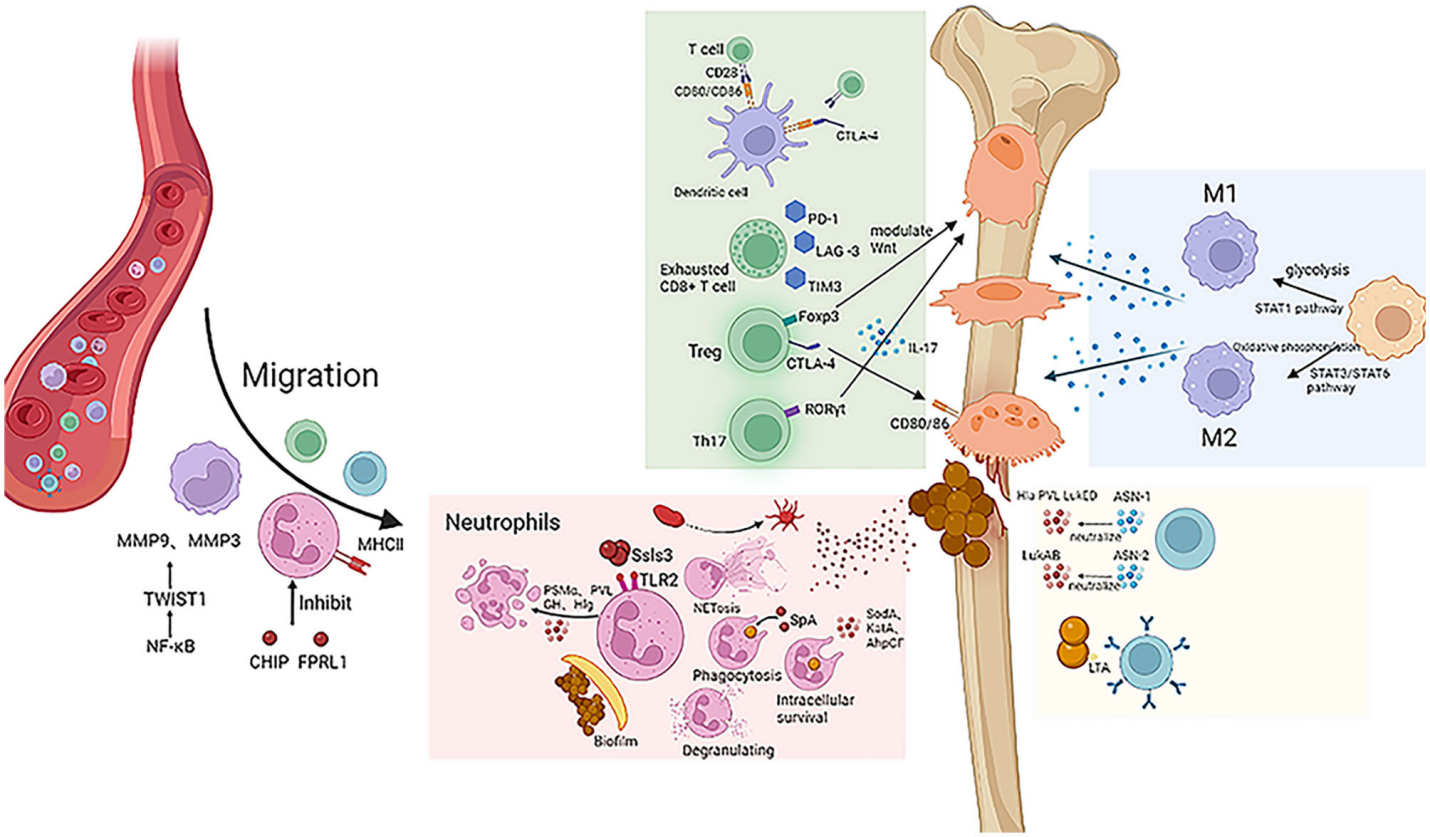
*S. aureus* exerts a negative effect on the host immune system. Macrophage migration is mediated by MMP9 and MMP3, and neutrophils are inhibited by virulence factors CHIP and FPRL1. *S. aureus* escapes the immune effects of neutrophils by biofilm formation, NET formation, and intracellular survival. Different oxygen and nutrient conditions can cause macrophages to differentiate into different cell subtypes and thus have different effects on inflammation. Checkpoints such as CTLA-4, PD-1, LAG-3, and TIM-3 inhibit T cell immune effect. Antibodies ASN-1 and ASN-2 can neutralize virulence factors such as Hla, PVL, and LukED.

*Staphylococcus aureus* (*S. aureus*) is the most common pathogen responsible for osteomyelitis and one of the common pathogens that cause a persistent infection (1). *S. aureus* disrupts the immune response in immune cells and mediates the balance between osteoclast and osteoblast, ultimately leading to prolonged infection and progressive bone destruction, which results in chronic osteomyelitis (2). *S. aureus* is now known to mediate chronic infection and bone destruction through the following mechanisms: (1) secretion of virulence factors (**Figure 1**)—These virulence factors secreted by *S. aureus* enhance bacterial colonization, subsequently inducing the death of immune cells and altering the immune environment, which leads to immune escape. It has also been shown that these virulence factors are closely associated with abscess formation in osteomyelitis (3, 4); (2) biofilm formation—Vascular damage and oxygen concentration reduction provide a good environment for biofilm formation by *S. aureus*. A biofilm not only acts as a physical barrier to prevent immune cells from contact with the pathogen (5) but also disables neutrophils to be activated and promotes macrophages to differentiate; (3) altered metabolism—Under infection, immune cells consume more energy, exerting a stronger immune effect, to compete with pathogens, and therefore pathogens alter their metabolism accordingly in order to survive (6, 7); (4) achieving intracellular survival—After engulfment by the immune cell, *S. aureus* is able to escape from the phagosome, sustain intracellular survival, and initiate intracellular replication (4, 8).

Meanwhile, *S. aureus* penetrates into the cortical bone and infects osteocytes. These infected osteocytes secrete more chemokines such as CCL5, CXCL1, CXCL8, CXCL9, CXCL1, and CXCL11, which activate neutrophils and T lymphocytes, promoting inflammation and bone destruction (9). *S. aureus* is also able to survive in osteoblasts, causing morphological changes or even cell death therein. Similarly, infected osteoblasts secrete more inflammatory factors to promote immune cell migration and enhance osteoclastogenesis. These osteoclasts increase their nuclear volume by two folds and their cell surface area by four folds, which greatly enhances their ability of bone resorption and ultimately leads to bone destruction (2, 10).

Since *S. aureus* disrupts the function of immune cells in osteomyelitis, immunotherapy has a great potential role in the treatment of osteomyelitis. The mechanisms of how *S. aureus* targets on immune cells and new immunotherapies for the treatment of osteomyelitis are currently under investigation. The aim of this review is to provide an overview of the effects of *S. aureus* on immune cells in innate and adaptive immune during the pathogenesis of osteomyelitis.

## 2 Innate immunity

### 2.1 Neutrophils

In general, in the early stages of infection, neutrophils are the first immune cells to respond to bacterial infection and exhibit their immune function at the infectious site. The mechanisms of their immune effects include (1) the production of reactive oxygen species to kill pathogens directly, (2) the production of antimicrobial peptides such as cathelicidin, lysozyme, and azurocidin by intracellular phagosomes, (3) the production of proteases to degrade bacterial proteins such as cathelicidin G, elastase, gelatinase, and collagenase, and (4) the formation of extracellular traps to capture and kill pathogens (11).

However, in *S. aureus*-infected osteomyelitis, chemotactic migration, activation, and phagocytic killing are disturbed, which inhibits neutrophil extracellular trap (NET) formation and impairs bacteria-killing ability. Neutrophils even undergo changes during infection, which induces tissue destruction and assists the bacteria in surviving and spreading at the infectious site.

#### 2.1.1 Chemotaxis

In the early stages of infection, host cells secrete large amounts of chemokines that recruit neutrophils to the infectious site and exert their bactericidal effects. CHIPs and FPRL1 inhibitory protein (FLIPr) are two virulence factors secreted by *S. aureus* which affect neutrophils’ chemotaxis function in osteomyelitis. Chemotaxis inhibitory proteins (CHIPs) reduce chemokine production and reduce neutrophil chemotaxis toward the infectious site (12). FLIPr inhibits formyl peptide receptor-like-1 (FPRL1) by decreasing intracellular calcium mobilization and facilitates neutrophil chemotaxis to the infectious site. In this condition, neutrophils are unable to arrive at the infectious site and thus cannot kill pathogens through reactive oxygen species and NETs (13). Thus, the reduced chemotactic capacity of neutrophils leads to a much reduced bactericidal effect of innate immunity. Therefore, targeting CHIPs and FLIPr secreted by *S. aureus* would be a new strategy to enhance neutrophil chemotaxis.

It has been well known that the life span of neutrophils is prolonged in patients with sepsis or severe bacterial infections (14), and Wagner et al. demonstrated that these prolonged neutrophils still produce superoxide and are equipped phagocytic activity, but their chemotaxis is significantly reduced (15). The team later showed that the neutrophil expression of the activation-related surface receptors including CD14 and CD64 remained upregulated during persistent infection, while the chemoattraction-related adhesion molecule CD62L was reduced, which may explain the lack of neutrophil chemotaxis in persistent infection (16).

#### 2.1.2 Antigen recognition and activation

In general, neutrophil activation depends on the recognition of pathogen surface receptors. Staphylococcal superantigen-like 3 (Ssl3) belongs to the staphylococcal-like protein (Ssls) family and is also a virulence factor secreted by *S. aureus*, which blocks pathogen recognition by neutrophils and inhibits their activation by competitively binding to Toll-like receptor 2 (TLR2) (17, 18); the virulence factor CHIPs bind competitively to complement C5a, preventing C5a from activating neutrophils after binding to the complement receptor (3). As a response, neutrophils secret inhibitory neutrophil elastase proteinase-3 and cathepsin G to digest CHIPs. However, *S. aureus* is able to produce extracellular adherence protein (Eap) and Eap homologs (EapH1 and EapH2), which, in turn, inhibit these neutrophil elastases (19). Moreover, during persistent infection, some neutrophils express MHC class II, which is able to present antigen to T cells. It has been demonstrated that such neutrophils in chronic infection have the capacity to present antigen and activate T cells (16).

A distinctive feature of *S. aureus* in osteomyelitis is the ability to form biofilms on the surface of the implant. Generally, without the shelter of biofilm, planktonic bacteria are quickly recognized and captured by the immune cells. Biofilms are formed when planktonic bacteria adhere to the surface of implants (e.g., internal fixations: plates, screws, etc.) (5). Immature biofilms cannot serve as a perfect barrier and can be penetrated by neutrophils, which are fully activated to perform their immune function. However, once the biofilm is mature, its function as a physical barrier to immune cells is greatly enhanced. Extracellular polymers (EPS) are a major component of biofilms and are the main immunosuppressive effector. EPS impede the recognition of bacteria by shifting the recognition target of neutrophils from the pathogen to itself, thus helping the pathogen to evade the host’s immune response and persist at the infectious site (20, 21). However, extracellular DNA (eDNA), one of the components of EPS, has the ability to activate neutrophils. It is most likely that the activation of neutrophils by eDNA is not dependent on TLR; surface molecules of neutrophils are possibly able to directly sense eDNA in the bacterial microenvironment and are then activated (22). It is clear that this important component of EPS has a completely different effect on immune cells than EPS. However, whether there are other components of EPS that affect immune cell function remains to be investigated in the future.

#### 2.1.3 Phagocytosis and killing

Neutrophils are the first line of defense for innate immunity and are usually involved in the immune response through phagocytosis and internalization, activation of degradative enzymes and cationic peptides in granules, extracellular trap formation, and antigen presentation (16, 23).

Staphylococcal complement inhibitor (SCIN) secreted by *S. aureus* suppresses neutrophil activation by inhibiting C3 convertase activity, which results in reduced complement C3 production as well as suppressed opsonization and phagocytosis in neutrophils (24, 25). As another virulence factor, Ssls7 inhibits the function of chemotaxis and complements production, pathogen phagocytosis and reactive oxygen species production (26, 27). Besides that, many other virulence factors are also involved in the inhibition of phagocytosis and killing of neutrophils. *S. aureus* protein A (SpA) inhibits antigen phagocytosis; alpha-type phenol-soluble modulin (PSMα), pantone viren albumin (PVL), albumin GH (LukGH), and gamma hemolysin (Hlg) induce neutrophil lysis and apoptosis and reduce the number of neutrophils (3).

The persistent intracellular survival of *S. aureus* is a crucial reason why neutrophils cannot play a full role in phagocytosis and killing. *S. aureus* interferes with the function of the neutrophil phagosome and thus persists in immune cells. The virulence factors superoxide dismutase (SodA and SodM), catalase (KatA), alkyl peroxide reductase (AhpCF), and streptomycin (encoded by crtOPQMN) modulate the cytotoxic effects of ROS, allowing pathogens to persist in the phagosome and preventing their complete elimination (28–30); the virulence factor Hla attenuates ROS-mediated killing by blocking the recruitment of phagosome toward the mitochondria. In recent years, Neumann et al. have found that *S. aureus* was usually labeled and killed in LC3-positive phagosomes, whereas *S. aureus* cannot be a target in phagosomes without LC3 modification. Therefore, in osteomyelitis, it is likely that *S. aureus* survives in the phagosome by interfering with the expression of LC3-modified protein production, and it is necessary to investigate the mechanisms by which *S. aureus* affects LC3 expression in osteomyelitis; it may be a new strategy to effectively block these pathways and restore the function of the phagosome to kill pathogens (31).

#### 2.1.4 Tissue damage

The formation of extracellular traps is crucial for neutrophils to clear pathogens. NETs are released by neutrophils and are covered with histones, elastase, myeloperoxidase, and cathepsin G (32). The function of NETs is to capture and clear *S. aureus* at the infectious site at the expense of inducing damage to endothelial cells and other organs (33). Fabrizio Semeraro et al. demonstrated that histone proteins mediated platelet activation through TLR4 and TLR2 to promote blood coagulation and thrombin formation. The researchers proposed that this may be related to platelet-rich microthrombosis in sepsis models. We speculated that it was possible that the histones in NETs also play a key role in the formation of microthrombosis in chronic osteomyelitis, which hinders tissue repair and provides conditions for the growth of *S. aureus*. Therefore, NETs not only trap pathogens but also play an important role in tissue destruction in chronic osteomyelitis (34).

In summary, *S. aureus* exerts a series of negative effects on neutrophils to escape immune reactions. Inflammatory factors like IFN-γ, EPS, and NETs are involved in such process, which made all of them become possible therapeutic targets. It is worth noting that virulence factors almost affect the overall process, especially in phagocytosis and killing. Therefore, how to clear virulence factors secreted by *S. aureus* remains to be investigated.

### 2.2 Macrophages

The function of macrophages is closely corresponding to the subtype they exhibit. By altering the subtype of macrophages, *S. aureus* mediates not only chemotaxis and phagocytosis in osteomyelitis but also osteogenesis and bone destruction.

#### 2.2.1 Subtype transition

In the early stages of infection, circulating monocytes are differentiated into macrophages by inflammatory cytokines. M1 macrophage is able to kill pathogens by releasing lysosomal enzymes and promoting inflammation by secreting pro-inflammatory factors. M2 macrophage secretes anti-inflammatory factors to inhibit excessive inflammation and produces platelet-derived growth factor, fibroblast growth factor, etc., to promote tissue repair (35). In chronic osteomyelitis, the interconversion and percentage between the two subtypes of macrophages are abnormal and imbalanced. The persistent presence of M1 macrophages induces an excessive inflammatory response, which may lead to severe tissue destruction. Likewise, the excessive production of M2 macrophages leads to insufficient phagocytosis and cytotoxic effects, biofilm formation, and bacterial resistance (36). It indicates that the mechanisms of macrophage action in chronic osteomyelitis are complex.

The STAT3/STAT6 pathway promotes macrophage polarization toward the M2 phenotype, whereas the STAT1 pathway promotes macrophage differentiation to the M1 phenotype. This suggests that the STAT3/STAT6 pathway negatively mediates the pathogen clearance ability in macrophages, which induces the persistence of osteomyelitis. Furthermore, it has been shown that IL-10 can trigger the STAT3 pathway to suppress the immune response of macrophages. Therefore, targeting this signaling pathway in macrophage may be a new immunoregulatory therapy for treating chronic osteomyelitis (37–39).

The biofilm formed by *S. aureus* is also able to influence macrophage polarization. When biofilms are not yet formed, the metabolic pattern of host immune cells is mainly based on glycolysis, which is able to participate in macrophage-mediated pro-inflammatory responses and contribute to the conversion of macrophages to the M1 phenotype. In contrast, when biofilms are formed, bacterial populations are significantly higher; thus, glucose and nutrients in the microenvironment are barren. Inefficient energy-producing pathways force host immune cells to choose oxidative phosphorylation to support macrophage conversion to M2 phenotype to repair damaged tissues (40, 41). Therefore, targeting mature biofilms formed by *S. aureus* may be a new strategy to modulate the excessive inflammatory response and tissue destruction caused by an imbalance in the ratio of M1 and M2 phenotypes of macrophages in osteomyelitis.

#### 2.2.2 Chemotaxis

Like neutrophils, macrophages are recruited by chemokines and leave the circulation to the infectious sites. In chronic osteomyelitis, macrophage migration is regulated by the NF-κB/TWIST1 signaling pathway. NF-κB induces the upregulation of TWIST1 expression to enhance macrophages polarized to M1 phenotype and migrate to the infectious site (36). In addition, TWIST1 induced the expression of MMP9 and MMP3, which can also promote macrophage migration (42).

#### 2.2.3 Phagocytosis and killing

Macrophages exert their immune effects mainly through pathogen phagocytosis. Macrophages are regulated by the PI3K/Akt-Beclin signaling pathway when they exert phagocytosis and killing under osteomyelitis conditions.

The PI3K/Akt-Beclin signaling pathway regulates macrophage autophagy, pathogen phagocytosis, and NF-kB-mediated inflammatory responses in *S. aureus* osteomyelitis. Recently, studies have shown that the PI3K inhibitors inhibited macrophage autophagy and impaired the phagocytosis of *S. aureus*. Meanwhile, when Beclin1 was knocked out and autophagy was inhibited, the NF-κB signaling pathway was activated, and a significant increase in inflammatory factors, such as TNF-α and IL-1β, can be detected in macrophages (43). It is clear that PI3K/Akt-Beclin positively regulates macrophage autophagy and phagocytosis and negatively regulates NF-κB-mediated TNF-α and IL-1β production in macrophages.

Obviously, some signaling pathways and relative transcription factors play key roles in macrophage differentiation, chemotaxis, and phagocytosis. It is important for us to explain the relationship among these signaling pathways, *S. aureus*, and macrophages. Mediating these pathways may become a new therapy in osteomyelitis treatment.

#### 2.2.4 Bone and macrophage

Bone formation, a dynamic process, is mediated by osteoblast-mediated bone formation and osteoclast-mediated bone resorption (20). Many studies have shown that macrophages were involved in bone formation and bone resorption in inflammation by regulating the functions of osteoblasts and osteoclasts. In chronic osteomyelitis, the balance of bone remodeling is disrupted, osteoblasts differentiation is inhibited, and more osteoclast precursor cells differentiate into osteoclasts, of which macrophages are significantly relevant (10, 44, 45).

##### 2.2.4.1 Osteogenesis

M2 macrophages promote the differentiation of mesenchymal stem cells (MSCs) into osteoblasts to repair damaged tissue (46). Furthermore, the osteogenic zone of long bones in mice with depleted macrophages is shorter and thinner than in normal mice, suggesting that the osteogenesis of osteoblasts is inextricably linked to macrophages (47).

BMP2, a member of the TGF-β family, is one of the first cytokines to be involved in the differentiation of mesenchymal stem cells into osteoblasts. BMP2 increases the expression of the osteogenic pathway by regulating key transcription factors, RUNX2 and ALP, in the osteogenic differentiation pathway, ultimately promoting osteoblast differentiation (48).

TNF-α is also an important factor that influences the differentiation of MSCs toward osteoblasts. It is secreted by M1-type macrophages and is a double-edged sword in the regulation of osteoblastogenesis. Its effect on osteoblasts depends on the dosage. Under physiological conditions, TNF-α promotes the migration and proliferation of osteoblasts by activating the p38/MAPK pathway (49). In contrast, under acute inflammatory conditions, excessive TNF-α inhibits osteoblastogenesis by inhibiting the Wnt pathway by regulating GSK3β (50). In addition, TNF-α affects osteoblasts by mediating miRNAs. Specifically, TNF-α-mediated miRNA-23b binds directly to the transcription factor RUNX2, negatively regulating RUNX2 and inhibiting downstream responses (47, 51). However, further research on how miRNAs recognize specific gene sequences on RUNX2 still remains to be discussed.

Oncostatin M (OSM), the IL-6 family cytokines secreted by macrophages, can also induce osteogenic differentiation. OSM binds to two kinds of mesenchymal stem cell surface receptors, the OSM receptors (OSMRs) and the leukemia inhibitory factor receptors (LIFRs), and regulates the JAK/STAT3 pathway to promote osteoblast differentiation (52).

GPNMB is a group of glycoproteins which are expressed in macrophages, especially in M2 subtype (53). It has been shown that GPNMB recruited MSCs to damage sites and stimulated osteoblastogenesis via the ERK/AKT and JAK/STAT3 pathways. However, other studies have also shown that excess GPNMB may stimulate osteoclast activity and induce bone loss (46).

Exosomes secreted by macrophages are also involved in the transformation of mesenchymal stem cells. Exosomes are vesicle-like bodies containing complicated ribonucleic acids and proteins, mediating cellular paracrine secretion and regulating cell function and intercellular communication. Exosomes secreted by macrophages contain CD9^+^, CD63^+^, CD81^+^, Tsgl01^+^, and Hsp70, which are internalized by MSCs and induce their differentiation toward osteoblasts (54).

In recent years, β-tricalcium phosphate (β-TCP) has been shown to activate the immune reaction of M2 macrophages. β-TCP stimulates the osteogenic differentiation of MSCs via the BMP2 pathway and subsequently activates distal-less homeobox 5 and the expression of transcription factors Runt-related transcription factors 2 (55). Furthermore, this study indicated that β-TCP was an ideal potential biomaterial for filling bone defects and had great promise for application.

In summary, these osteogenic molecules and cytokines, which are secreted by macrophages, may be affected by *S. aureus* during osteomyelitis. Its production and secretion processes may be inhibited or altered in osteomyelitis, which ultimately prevents the normal regulation of osteoblast differentiation and impairs osteogenesis.

##### 2.2.4.2 Bone resorption

Osteoclasts derived from the monocyte/macrophage lineage are homologous to macrophages in bone tissue (36, 38, 56). Osteoclast precursor cells differentiate into mature osteoclasts which subsequently mediate bone resorption through various signaling pathways. The main signaling pathway involved in infection is the RANKL/RANK pathway. Bone defects are very common in post-traumatic osteomyelitis. Osteoclasts are directly associated with bone defects, and macrophages are able to participate in the regulation of osteoclastogenesis. M1 macrophages secrete large amounts of inflammatory cytokines, including TNF-α, IL-6, and INF-γ, which promote osteoclastogenesis (57, 58).

TNF-α has been recognized as the central cytokine affecting the RANKL/RANK signaling pathway. TNF-α not only regulates osteoblast function, which indirectly impairs bone formation (57), but also mediates osteoclast production, which directly causes bone resorption. In the presence of large amounts of inflammatory cytokines, TNF-α stimulates osteoblasts to secret RANKL and induces stem cells to secret M-CSF, which can lead to more frequent and tighter binding of RANKL and RANK (59). TRAF6 is the switch for this pathway. TNF-α enhances the RANKL-TRAF6 signaling pathway through TRAF2 (60). However, whether TNF-α must be present in the presence of RANKL to enhance this signaling pathway is still controversial. Most researchers support the notion that TNF-α relies on RANKL to exert its effects, while others take the opposite view (61, 62). In addition, some studies have also shown that TNF-α is able to regulate the growth of osteoclasts in the absence of RANKL (63). In recent years, it has been shown that TNF-α exerts a pro-differentiation effect on osteoclast precursor cells via sialylation. Sialic acid is a neuraminic acid derivative involved in osteoclastogenesis. TNF-α triggers the differentiation of highly sialic-acidified precursor osteoclasts, but such effect of TNF-α is diminished when the sialylation of precursor osteoclasts is disturbed (64). This suggested that TNF-α may not need to rely on RANKL to promote osteoclastogenesis.

## 3 Adaptive immunity

### 3.1 T cells

After antigen activation, T lymphocyte cells are generally differentiated into helper T cells (CD4^+^T cells) and cytotoxic T cells (CD8^+^T cells). CD4^+^T cells promote immune responses by secreting different inflammatory factors to activate other immune cells; sometimes they also control the progression of inflammation by inhibiting the proliferation of immune cells. CD8^+^T cells kill pathogens directly by secreting proteins and cytokines, such as perforin and granzyme (20).

#### 3.1.1 Antigen recognition and activation

Most dendritic cells are able to activate T cells by presenting antigens. Leukocidin AB (LukAB), a virulence factor secreted by *S. aureus*, prevents T cells from being activated by killing dendritic cells, which play a key role in antigen presentation (65).

Cytotoxic T lymphocyte A antigen 4 (CTLA-4) is secreted primarily by T cells and is an inhibitory molecule that binds to CD80/CD86 on the surface of antigen-presenting cells, preventing CD80/CD86 from binding to CD28 on the surface of T cells. It thereby interferes with the activation of APCs on T cells and blocks pathogen clearance, leading to infection persistence (20, 66).

#### 3.1.2 Pathogen killing

During the transition from the acute to the chronic phase of osteomyelitis, the function of CD8^+^T cells is impaired in this process and then becomes a participant in the development of chronic osteomyelitis.

It has been found that during chronic osteomyelitis, the killing capacity of cytotoxic T cells was greatly diminished in response to the sustained stimulation of large amounts of antigens and inflammatory factors. These T cells with immunosuppressive properties are called exhausted CD8^+^T cells (66). Exhausted CD8^+^T cells produce fewer cytokines such as IFN-γ, TNF-α, IL-12, IL-18, and IL-12. At the same time, the production of cytotoxic effectors such as granzyme and perforin is also reduced, which causes the inefficient elimination of pathogens (67). Importantly, exhausted CD8^+^T cells express various suppressor receptors such as PD-1, LAG-3, 2B4, CD160, TIM3, and TIGHT (67–69). These suppressor receptors continue to exert immunosuppressive effects in persistent infections. Although the immunosuppressive effect of a single inhibitory receptor is limited, exhausted T cells often express multiple inhibitory receptors, such as PD-1 and LAG-3, which synergistically exert a more potent immunosuppressive effect than a single receptor (70). In addition, the metabolic status of exhausted CD8^+^T cells is altered to adapt to the environment of chronic infection. Normal CD8^+^T cells rely primarily on glycolysis for energy (71, 72); however, exhausted CD8^+^T cells suppress the activation of AKT in the energy synthesis pathway as well as mTOR activity by CTLA-4 or PD-1 (73, 74), resulting in a shift to fatty acid metabolism for energy. It is compatible with the environment of chronic infection that allows the cells to survive in nutrient-deficient environments (75, 76). This suggests that reversing the change in the energy synthesis pathway in CD8^+^T cells or inhibiting fatty acid metabolism in CD8^+^T cells could be a new immunotherapy prospect to reduce the number of exhausted CD8^+^T cells and rescue immunosuppression statutes.

Recently, studies have shown that a difference in transcription factor expression exists between normal effector CD8^+^T cells and exhausted CD8^+^T cells. In recent years, transcription factors are considered to be closely associated with this functional transformation of CD8^+^T cells, including T-bet, EOMES, and TCF1.

T-bet and EOMES are a pair of transcription factors that influence the differentiation fate of initial CD8^+^T cells. When initial CD8^+^T cells express more T-bet than EOMES, they will differentiate toward terminal effector CD8^+^T cells. Conversely, if EOMES are expressed more, initial CD8^+^T cells will differentiate toward memory T cells. The continued high expression of EOMES leads to progressive CD8^+^T cell exhaustion (77). Some researchers believe that a persistent high EOMES expression may be associated with high PD-1 expression, which has been shown in upregulation during chronic osteomyelitis (70, 78).

TCF1 is a T cell-specific transcription factor that is thought to be essential for the differentiation and maintenance of exhausted CD8^+^T. In the early stages of chronic infection, TCF1 is co-expressed with CXCL5 in CD8^+^T cells, then T cells gradually exhibit an exhausted CD8^+^T cell profile. CD8^+^T cells with TCF1 knockdown show a tendency to differentiate toward effector CD8^^+^^T cells (79, 80). PD-1 expression had a positive effect on TCF1 expression. Thus, the expression of T-bet, EOMES, and TCF-1 on exhausted CD8^+^T cells was all closely associated with PD-1 (81). However, the specific mechanisms by which these transcription factors affect CD8^+^ T cells and the mechanisms by which PD-1 interacts with T-bet, EOMES, and TCF1 remain unclear. It has been reported that CTLA-4 secreted by Treg is able to disrupt the CD80/PD-L1 heterodimer on antigen-presenting cells, resulting in an increase of free PD-L1 (66). Therefore, we speculate that increased PD-L1 activates more PD-L1/PD-1 signaling in osteomyelitis, which may be involved in regulating EOMES expression and mediating the generation of exhausted CD8^+^T cells.

Th17 and Treg cells and Th17/Treg-related immunosuppression are strongly associated with osteomyelitis persistence and bone destruction. Th17 cells, with RORγt on its surface as a unique marker, promote inflammation and inhibit osteoclastogenesis. The Treg surface marker is Foxp3, which is also a transcription factor, and deletion of Foxp3 leads to a reduction in the number of Treg cells. Treg negatively regulates the inflammatory response and also promotes osteoclastogenesis (13, 82–84).

*S. aureus* is able to activate Treg cells to suppress the host immune response. Sophia Björkander et al. demonstrated that *S. aureus* activated T cells via superantigens, inducing the large secretion of cytokines in T cells, which activate Treg cells, thus suppressing the immune response (85).

Th17 cells are differentiated from the initial T cells. TNF-α and other inflammatory factors, such as IL-1, IL-6, IL-21, and IL-23, enhance RORγt expression and inhibit Foxp3 expression, which induces the differentiation of initial T cells to Th17 cells (83). Thus, we believe that affecting the T cell differentiation pathway by blocking the secretion of TNF-α, IL-1, and IL-6 can be a strategy to control the inflammation and tissue destruction induced by Th17 cells.

In conclusion, different subtypes of T cells may play different roles in osteomyelitis. CD4^+^T cells interfere with normal immune reaction through the expression of CTLA-4; therefore, how *S. aureus* affects the T cell expression of CTLA-4 and whether its virulence factors have an impact on CTLA-4 expression in T cells are critical to the study of a new therapeutic target in osteomyelitis. CD8^+^T cells turn into exhausted CD8^+^T cells in osteomyelitis. T-bet, EOMES, and TCF1 are some crucial factors that are associated with an attenuated immune reaction. Treg cells and Th17 cells have the opposite effect, and how to mediate their function in osteomyelitis remains to be explored.

#### 3.1.3 Bone destruction

Th17 and Treg cells not only have opposite immune effects in the inflammatory process but also play distinct roles in bone production and destruction.

Treg cells inhibit osteoclast production and thus reduces bone destruction in osteomyelitis, but the mechanism for this remains controversial. Some studies believe that Treg cell inhibits osteoclast production mainly through decreasing the secretion of TGF-β, IL-4, and IL-10 (83). Other studies suggest that Treg cells inhibit osteoclastogenesis primarily through CTLA4-dependent intercellular contacts to reduce bone destruction. Treg-derived CTLA-4 binds to B7-1/B7-2 (CD80/86) on the surface of osteoclast precursors and impairs their differentiation to osteoclasts (86). However, Abdul et al. demonstrated that Treg cells also promoted osteoblastogenesis to accelerate bone remodeling (84). The Wnt signaling pathway is a pathway that regulates osteoblastogenesis. Wnt10b, a ligand for Wnt, is a potent enhancer of the regulation of osteoblast proliferation. Under butyrate conditions produced by *Lactobacillus*, Treg cells are involved in regulating the activation of the Wnt10b promoter, thus promoting Wnt10b expression (84).

Th17 cells mediate the formation of osteoclasts in various ways. First, the cytokine IL-17 secreted by Th17 cells not only directly increases the expression of RANKL in osteoblasts but also induces the secretion of IL-6, TNF-α, and IL-1, promoting the expression and activation of RANKL in osteoblasts. In addition, Th17 cells can also secrete RANKL (87). In conclusion, osteoblastogenesis is regulated by Th17 cells and promotes bone destruction during inflammation.

Therefore, several cytokines like CTLA-4, IL-17, and IL-6 and signaling pathways like Wnt and RANKL/RANK are involved in the process of Tregs cells and Th17 cells mediating osteoclastogenesis, but other cytokines and pathways in this process are unknown and still remain to be found out. In this review, we figure out the relationship among these factors.

Th17 cells promote inflammatory response and bone destruction, whereas Treg cell inhibits the degree of inflammation and promotes bone formation. Actually, the Th17/Treg ratio is modulated by different conditions. When intense inflammation occurs, inflammatory cells secrete large amounts of inflammatory factors such as IL-6. Inflammatory factors represented by IL-6 stimulate the production of Th17 cells via the STAT3 pathway, whereas in the absence of IL-6, the production of Th17 cell is inhibited (88–90).

Hypoxia-inducible factor α (HIF1α) affects Th17/Treg ratio in a hypoxic environment (91). HIF1α not only increases RORγt expression to promote Th17 cell differentiation but also inhibits Foxp3 expression via the proteasomal degradation pathway under hypoxic conditions, resulting in a relative increase in RORγt expression (92). In summary, IL-6 and HIF1-α, which are produced under hypoxic conditions caused by intense inflammation, regulate Th17/Treg ratio through different pathways, implying that more Th17 cells are induced in an intense inflammation-related hypoxic environment. Conversely, in a more mild, chronic, normoxic inflammatory environment, initial T cells are more inclined to differentiate into Treg cells. Therefore, an ideal environment is critical to balance the Th17/Treg ratio.

### 3.2 B cell

There are two kinds of antibodies (ASN-1 and ASN-2) secreted by B lymphocytes which can neutralize the virulence factors secreted by *S. aureus*. ASN-1 neutralizes alpha-hemolysin (Hla), panton-valentine leucocidin, leukocidin ED (LukED), and g-hemolysin, while ASN-2 neutralizes LukAB/LukGH (93). Thomsen et al. collected peripheral blood from a 12-year-old boy with *S. aureus*-infected osteomyelitis, isolated B cells, and three kinds of monoclonal antibodies. They demonstrated that SA-13, SA-15, and SA-17 had the capacity to effectively reduce the damage of immune cell function caused by LukAB in the acute phase (94). However, *S. aureus* also has an immune escape mechanism to humoral immunity. The virulence factor SpA inhibits antibody-mediated phagocytosis by binding to antibodies as well as causes B cell apoptosis (95–97). *Staphylococcus aureus* enzyme (Sak) causes IgG cleavage and degradation that greatly diminishes IgG-mediated phagocytosis by neutrophils (98). In addition, Pelzek et al. showed that, although patients had high levels of antibodies in acute phage, the antibodies’ response disappeared at 6 weeks of follow-up visit. These results suggest that *S. aureus* infection after acute phage is not sufficient to induce secondary recall responses that result in persistently elevated antibody levels. Therefore, we speculated that although B cells were capable of generating many antibodies against *S. aureus* virulence factors, the development of chronic osteomyelitis was related to the failure of B cells to keep the level of antibody generation (99).

B cells can be activated by LPS. However, as a cell membrane component of gram-positive bacteria which is similar to LPS, lipoteichoic acid (LTA) is capable of inhibiting B cell proliferation and interfering with the LPS-induced B cell immune response. Although it is difficult to determine whether LTA is involved in the immunosuppressive effects of *S. aureus* on B cells, LTA does provide us with a new perspective to explore the negative immune role of *S. aureus* in osteomyelitis (100).

Zeng et al. showed that, in periodontitis, B cells secreted pro-inflammatory cytokines, receptor activator of NF-κB ligand (RANKL), matrix metalloproteinases, and autoantibodies (Abs) to mediate inflammation as well as bone destruction (101, 102). Regulatory B cells (Bregs), a subtype of B cells, play an opposite role; Bregs produce anti-inflammatory cytokines, such as IL-10, TGF-β, and IL-35, which inhibit the development of inflammation (103, 104), but it is unclear whether Bregs display an opposite role in bone remodeling.

In conclusion, B cells are likely to be involved in the chronicity of osteomyelitis and bone destruction in *S. aureus* infection just like other immune cells.

## 4 Immunotherapy targets

Antibiotic therapy with surgical debridement is a common strategy to treat osteomyelitis in the clinic. However, in Wu and team’s investigation, they review the osteomyelitis patients in their clinical center during 2013 to 2020 and show that, of 482 patients, 13.7% presented with infection persistence after initial debridement and antibiotic treatment (6 weeks), thus needed repeated debridement, 8.5% had recurrence after all treatment ends and a period of infection cure, and in 3.5% of patients, complications were observed (105). Some osteomyelitis patients face many problems such as more postoperative complications and high cost of follow-up treatment. In addition, patients with osteomyelitis have a very high rate of re-infection after surgery (15%–40%). Finally, they have to replace the internal fixation (106). Therefore, it is urgent for us to study a new therapy to supply traditional antibiotic treatment. In fact, immunotherapy has been applied in tumor and autoimmune disease treatment. Moreover, there is a similar immune suppression between a tumor and an infectious microenvironment, so we believe that immunotherapy can be used to treat infectious disease (107).

During the investigation on clinical immunotherapy of chronic osteomyelitis, we still have to figure out many complex mechanisms, including functional redundancy among virulence factors, differential expression of virulence factors during different stages of growth, or heterogeneity in protein expression throughout the bacterial biofilm (108).

Harro et al. have shown that they developed a biofilm-specific pentavalent vaccine. In the mice model, the mortality rate of mice immunized with the pentavalent vaccine after an injection of *S. aureus* was 16.7%, while that of the control group was 91.7%. The complete clearance of infection in the pentavalent vaccine group was 66.7%, while that in the control group was only 8.3%, suggesting that the pentavalent vaccine targeting biofilms may have preventive and immune effects in the clinical treatment of chronic osteomyelitis (109).

Actually, vaccine targeting neutralization of *S. aureus* virulence factors has also been investigated in other diseases. Some kinds of anti-toxin monoclonal antibodies are used in respiratory-infected clinical trials—for example, Kailasan’s team investigated that rabbit polyclonal antibodies against LukAB, α-toxin, and PVL, when combined together, nearly completely neutralize their cytolytic effect against human immune cells. Although there is no existing evidence indicating that such antibody therapy has the same effect in *S. aureus*-infected osteomyelitis, they deserve to be investigated as potential therapies for osteomyelitis.

In the past, many studies in immunotherapy focus on vaccine development; however, in recent years, the increased number of studies tried to focus on the restoration of immune cell function through various targets, such as immune checkpoints and transcription factors.

It was mentioned that PD-1 overexpression induces CD8^+^ T cell exhaustion. Therefore, PD-1 is the key for recovering the effective immune response of CD8^+^ T cells. At first, Deak et al. showed that IL-2 combined with checkpoint inhibitors prevented the stem-like PD-1^+^TCF-1^+^CD8^+^T cells from differentiating toward exhausted T cells. In a recent work, they demonstrated that muPD1-IL2v differentiated PD-1^+^TCF^+^ stem-like CD8^+^ T cells into better effector CD8^+^ T cells in tumor or chronic LCMV infection (110). Their finding inspires us that IL-2 or muPD1-IL2v may exert a similar effect to enhance the immune response in chronic osteomyelitis. In addition, IL-2/STAT5 was demonstrated to have a key role for the conversion of CD^+^4 T cells to Treg cells. IL-2 binds to the domain of STAT and then triggers Foxp3 and CTLA-4 expression (111). The high expression of CTLA4 has an important role in immunosuppressive effect in Treg cells; therefore, it is considered as a potential target of osteomyelitis treatment. Wuwei Xiaodu Drink (WWXDD) is a traditional Chinese medicine prescription which contains lonicera flower, chrysanthemum flower, dandelion, siemiaquilegia ruber, and herba violae. The clinical application of WWXDD in wound infections achieved substantial results (112). Moreover, Huang et al. believed that WWXDD had a similar result in osteomyelitis; thus, they established a chronic osteomyelitis model in rats. They demonstrated that luteolin, chryseriol, kaempferol, and quercetin are the main active compounds of WWXDD, which are capable for downregulating the percentage of Treg cells via IL-2/STAT5 and suppressing the increased level of Foxp3 and CTLA-4 in osteomyelitis (113). Therefore, there have been proper valuable evidence supporting that WWXDD may become a new option for clinicians to treat chronic osteomyelitis.

Obviously, biofilm is an essential factor for the formation of chronic infection; thus, it is necessary for us to find out effective solutions to clear such barriers, which is not limited to vaccination. Next Science wound gel is applied in wound healing and disinfection, which targets biofilm in wound. The antimicrobial components within the gel are able to effectively destroy the EPS matrix and lyse the bacteria encapsulated within the matrix (114). However, the results of the gel in osteomyelitis remain unclear. It is unsure whether the gel has the capacity to physically access biofilm attaching in deep-seated bone. Exebacase (Lysin CF-301), an antistaphylococcal lysin, possesses ideal efficiency to clear staphylococcal biofilm. It has been investigated that exebacase removed all biofilms formed on catheters within 60 min and killed all released bacteria by 6 h (115, 116). Exebacase not only destroys staphylococcal biofilm in chronic osteomyelitis but also has a rapid bactericidal effect. Karau et al. have shown that, in an acute methicillin-resistant *S. aureus* (MRSA) osteomyelitis model, exebacase has synergistic activity with daptomycin, which is able to penetrate bone tissue (117). Exebacase may improve the ability of daptomycin to bind to its targets (118). Thus, exebacase plus daptomycin is an effective combination for osteomyelitis, including acute and chronic stages. It has great therapeutic prospect in the clinical treatment of osteomyelitis.

It is clear that the RANKL/RANK pathway plays an important role in mediating osteoclastogenesis and bone resorption. Therefore, targeting this pathway to reduce the production of RANKL is able to inhibit osteoclastogenesis, which may be a potential treatment for bone defects in chronic osteomyelitis. Studies have demonstrated that bisphosphonate can be applied in osteoporosis and HIV infection-induced low bone mineral density by targeting RANKL-induced expression (119, 120). Huang et al. suggested that zoledronic acid, which is one of the bisphosphonates widely used in preventing bone loss, is able to inhibit osteoclast differentiation by inhibiting the RANKL/RANK/TRAF6 pathway and its downstream NF-κB and JNK signaling pathways (120). Another bisphosphonate, alendronate, is demonstrated to improve bone mineral density by decreasing the osteoclast activity in HIV-infected patients. Thus, it is possible for bisphosphonates to become a new way to treat bone destruction in osteomyelitis. However, Kobayashi et al. investigated the effect of bisphosphonates in chronic osteomyelitis by establishing rat models, and they show that, although bisphosphonates are able to inhibit osteoclast differentiation, they protect necrotic formation and thus provide suitable nidus to infection. Compared to bisphosphonate, anti-RANKL ab is less effective in inhibiting bone destruction; however, it is beneficial to the clearance of necrotic bone (121). Thus, anti-RANKL ab also has advantages in the treatment of osteomyelitis. More clinical results are expected to help clinicians make better clinical decisions.

Twist Family BHLH Transcription Factor 1 (TWIST1) is a critical apoptosis inhibitor in embryonic development, tumor metastasis, and initiation. However, it plays a protective role in the later stage of *S. aureus*-induced osteomyelitis such that it inhibits macrophage apoptosis by blocking the activation of the mitochondrial apoptosis signaling pathway (122). Wang et al. demonstrated that elevated TWIST1 protects macrophages from programmed cell death cascade induced by calcium overload, which inhibits the antimicrobial ability of *S. aureus*-induced osteomyelitis models (123). Therefore, TWIST1 is a potential target for immunotherapy. However, it still remains to be investigated how TWIST1 can modulate calcium overload and which signaling pathways are involved in this process.

*S. aureus* is generally divided into MRSA and methicillin-sensitive *S. aureus* (MSSA). MSSA is known as the most common pathogen of osteomyelitis (124); however, in recent years, the proportion of MRSA-infected osteomyelitis has gradually increased. In fact, MRSA-induced osteomyelitis is more likely to cause high fever, tachycardia, pain, and claudication (125). Hospital-associated MRSA (HA-MRSA) infects patients in hospitals, while community-acquired MRSA (CA-MRSA) infects patients without a recent hospitalization (126). CA-MRSA equips higher virulence, producing much more virulence factors. It was shown that SCCmec is a mobile genetic element. The structure of SCCmec in HA-MRSA and CA-MRSA is quite different (127). SCCmec of HA-MRSA includes the psm-mec gene, which associates with *S. aureus* colony spread and biofilm formation, and the strain without psm-mec gene produces more virulence factors than that which carries the psm-mec gene. Furthermore, Kaito et al. have shown that psm-mec RNA is able to modulate PSM-α, which is a cytolytic toxin of *S. aureus* (126). Therefore, we believe that the psm-mec gene is a key factor in reducing the virulence of *S. aureus* and may become a new target for the treatment of *S. aureus*-infected osteomyelitis.

## 5 Conclusion

Osteomyelitis is a type of chronic bone disease with bacterial infection which is commonly caused by open fractures or postoperative implant infection. Especially in patients who suffer from open fractures, they are not only confronted with tissue damage but also a bacterial infection that causes osteomyelitis. *S. aureus* is one of the most common pathogens of osteomyelitis, and there is no effective therapy yet for *S. aureus*-caused osteomyelitis.

In recent years, more and more studies have focused on immune cells in osteomyelitis. They try to disclose the mechanisms of how immune cells mediate osteomyelitis and how *S. aureus* altered immune cells. Moreover, the therapies which are basic on immune cells still remain to be investigated. Thus, this article gives an overview of the relationship between immune cells, *S. aureus*, and osteomyelitis. *S. aureus* impairs the immunity reaction by virulence factors, biofilm, and intracellular survival. Impaired immune reactions enhance acute infection transferring to the chronic stage. During chronic stage, the function of immune cells is altered by inflammatory factors, immune checkpoints, metabolism, and transcription factors. Meanwhile, immune cells are closely associated with osteoblastogenesis and osteoclastogenesis, which are involved in bone formation and bone resorption.

However, the molecular mechanism of immune cells in chronic osteomyelitis remains to be further studied. Moreover, the role of B cells in the development of osteomyelitis and their interaction with *S. aureus* have not been studied. Thus, it is necessary for us to investigate the mechanism of immune cell function in *S. aureus*-induced osteomyelitis and explore new immunotherapy for osteomyelitis.

## Author contributions

BY and GL conceived and designed the study. YC, ZLiu, and ZLin searched the literature. YC and ZLiu drafted the article. ML, YF, GL, and BY revised the manuscript. All authors contributed to the article and approved the submitted version.
